# Towards correlative archaeology of the human mind

**DOI:** 10.1515/hsz-2023-0199

**Published:** 2023-10-12

**Authors:** Lukasz Piszczek, Joanna Kaczanowska, Wulf Haubensak

**Affiliations:** Department of Neuronal Cell Biology, Center for Brain Research, Medical University of Vienna, A-Vienna, Austria; a:head bio, Dr.-Bohr-Gasse 7, VBC6, A-1030 Vienna, Austria; Research Institute of Molecular Pathology (IMP), Vienna Biocenter (VBC), Campus-Vienna-Biocenter 1, A-1030 Vienna, Austria

**Keywords:** evolution, archeology, genomic, neuroscience, cognition, hominins

## Abstract

Retracing human cognitive origins started out at the systems level with the top-down interpretation of archaeological records spanning from man-made artifacts to endocasts of ancient skulls. With emerging evolutionary genetics and organoid technologies, it is now possible to deconstruct evolutionary processes on a molecular/cellular level from the bottom-up by functionally testing archaic alleles in experimental models. The current challenge is to complement these approaches with novel strategies that allow a holistic reconstruction of evolutionary patterns across human cognitive domains. We argue that computational neuroarcheology can provide such a critical mesoscale framework at the brain network-level, linking molecular/cellular (bottom-up) to systems (top-down) level data for the correlative archeology of the human mind.

## 1 Introduction

We humans share an anthropocentric fascination for the origins of our mind, for what made us human, and what sets us apart from our closest relatives, both alive or extinct.

This quest poses several key goals. The first and foremost aim is to reconstruct both direct ancestry (species that are in the direct ancestral lineages, but are now extinct), and how recent human speciation differs from that of other archaic hominins (e.g., Denisovans and Neanderthals). Second objective is to extend these methods as far back in our lineage as possible which would include the events in distal primate evolutionary history, extending to ancient monkeys and apes. Finally, in order to pinpoint those traits that were key to human evolution, it is essential not only to chart species differences (e.g., in brain size, skeletal or physiological makeup), but also to distill those changes that led to adaptive evolution (i.e., those traits that were under functional selection).

To carry out such a task, it is paramount to overcome the inherent problem that cognitive evolution took place in ancient brains that are inaccessible for experimental exploration. Surveying the variety of tools for exploring human cognitive traits conjoins various multidisciplinary efforts including hands-on archaeology, extracting ancient genomes, experimental science (e.g., genetically modified animal and organoid models carrying human and/or archaic alleles) and in silico predictions, integrated across biological levels into ‘correlative archeology’. This can be achieved by linking top-down systems level and bottom-up molecular genetics/cellular level methods, with brain data science at the mesoscale level. This will ultimately provide an understanding of neurocognitive evolution across neurobiological levels, typically required for comprehensive mechanistic insight of brain function (Pfaff et al. 2019).

## 2 Top-down deconstruction of brain cognitive evolution

Exploring the human past from fossil and archeological records has been a historical and highly informative entry point into human cognitive evolution. In the late 1850s, the discovery of the Neanderthal and the realization that anatomically modern humans (AMHs) evolved from ape-like archaic ancestors almost immediately sparked the interest in identifying neuroanatomical (and cognitive) differences among the human evolutionary line. The most well-known top-down approach links fossil endocast patterns to comparative neuroanatomical and functional data from extant species, and in a second step, interpolates ancestral states along these lines, dating back as early as 20 million years ago (Mya) (Ni et al. 2019). These top-down interpretations, from size to function, suggest that region specific brain expansion might point towards accelerated evolution in specific regions and their associated functions. These patterns vary between the brain regions, with strongest neocortical acceleration earlier at haplorrhine origins (Miller et al. 2019). Comprehensive Bayesian phylogenetic neuroanatomy of both extant and fossil species identified directional and accelerating evolution towards larger brains as hominins diverged from other primates, and again as humans and Neanderthals diverged from other hominins. However, since reconstruction of the Neanderthal skull revealed a similar brain size to AMH, this suggests that encephalization alone does not make us special. Verily, endocast extrapolations from early primate evolution onward, suggest complex regional evolution, not easily predictable from basic scaling laws (Ni et al. 2019). This in turn demonstrates that fossil endocasts alone may not be sufficient for differentiating the complexity of higher cognitive traits under selection implicit in the archeological record.

**Figure 1:**
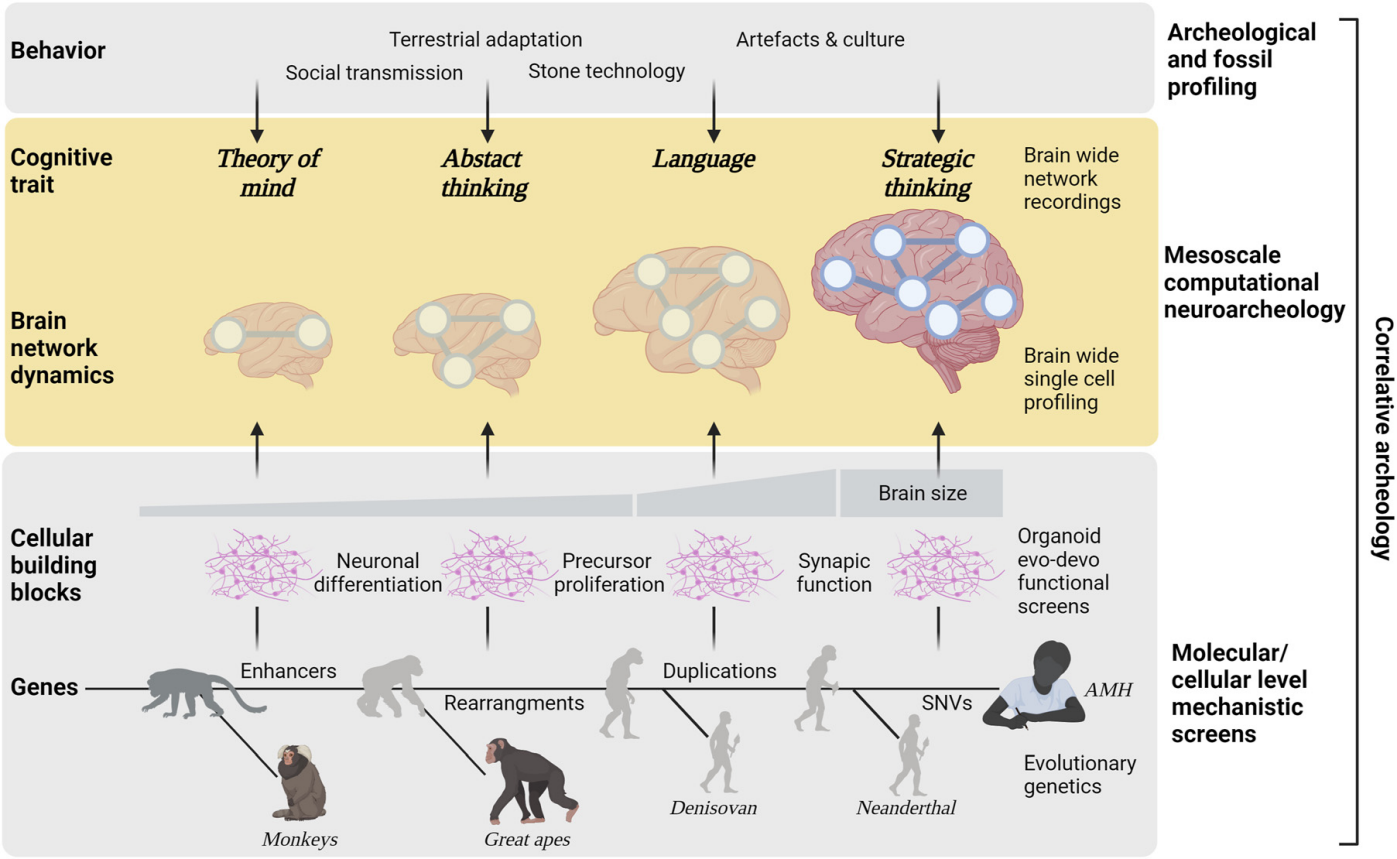
Multi-level integration in correlative archaeology of human mental traits. Mesoscale computational neuroanatomy links molecular/cellular level mechanistic insights to systems level cognitive traits and provides a framework for understanding human behavioral specialization inferred top-down from archeological records. Symbolic evolutionary timeline depicts exemplary scenarios of gene regulation and mutation that influence the cellular level functions (e.g., neuronal differentiation), and thus impact the functional networks (e.g., theory of mind, abstract thinking, language, strategic thinking) in human ancestry. These networks lay the foundation for behavior and mental states (the only effectors which leave direct traces in fossils and remains of mental and material culture). Linkage of the molecular changes with the behavior (for the entire ancestral line and extant species) is possible via correlating gene expression patterns in the functional network space – here human brain. Shaded brain networks depict the possibility of using other primate brain templates, for which large genetic and functional datasets will be available in the future. Created with BioRender.com.

Indeed, inferences of cognitive abilities from archeological traces of behavioral habits (hunting styles, burying rituals) and the creation of artifacts (such as tools and art) reveal a complex past (Figure 1). The earliest of such traces stem from Australopithecus up to 3.3 Mya and the usage of stone tools (Lewis and Harmand 2016), followed by an adaptation in life history strategy (Antón 2003) and hunting gathering styles (Domínguez-Rodrigo et al. 2014). A subsequent transition emerged when the tools evolved to their more complex forms, such as hand axes (by 500 Kya). This, together with the spread of controlled use of fire (starting around 800 Kya) (Goren-Inbar et al. 2004) as well as structured organization of workspace indicates general higher cognitive function and social skills required in hominin habitats (Alperson-Afil et al. 2009). These periods are again followed by phases reflected by increase in technological and demographic complexity, culminating in the creative explosion between 60 and 30 Kya (Foley and Mirazón Lahr 2011; Mithen 1996). With this, many art forms appeared, like decorated tools and pottery, and perhaps most famously cave paintings. Interestingly, the oldest known artifacts and cave paintings originated from Neanderthals and had similar abstraction as those attributed to AMHs (Hoffmann et al. 2018). Archeological traces from Neanderthal sites suggest that they also cared for their sick and buried their dead and ultimately shared many habits typically associated with AMHs. From this data, the evolution of specific cognitive traits can be inferred from the increase in the level of abstraction in the fields of mechanical understanding, sensorimotor control and visuo-spatial understanding that are required for the complex tool building process (Lewis and Harmand 2016). Likewise, increased social behavioral complexity suggests evolution in theory of mind and a transition in language skills (Corballis 2009). The period of encephalization with more coding capacity may have led to more fine-grained distributed depiction of episodic representations, with more coding capacity for detail. This in turn facilitated generalization and abstraction resulting in the cultural transmission of abstract concepts. In later phases, improved interaction between domain-(function) specific brain modules may have driven cognitive skills, such as associative and analytical thought, to further advance cultural evolution (Gabora 2007).

Overall, archeological data from either traces or remains, reflects human evolution at systems levels (brain size and behavior). However, it is difficult to pinpoint which brain functions were under adaptive selection at specific evolutionary time points in the human lineage. Given the scarcity of older records and the overlap in archeological profiles between hominins, it is hard to archaeologically differentiate particular domains important and perhaps unique to AMH. Thus, these archeological profiles of behavioral traces and brain size may serve as a reference framework, best supported by complementary methodologies for further exploration of the underlying evolutionary adaptations at the brain’s structural, functional and neurogenetic levels.

## 3 Bottom-up reconstruction of neurogenetic evolution

Complementing archeology, evolutionary genetics has revolutionized insights into the AMH history. First and foremost, it has allowed the study of events dating back more than 60 Mya of ancestral lineages. This power was potentiated by integrating paleogenetic data from archaic humans, ultimately facilitating sorting out phylogenetic and genomic differences to great apes (The Chimpanzee Sequencing and Analysis Consortium 2005) and the subsequent evolution of Neanderthal (Green et al. 2010) and Denisovan (Reich et al. 2010) relatives. Beyond the complex pattern of separation and interbreeding between these hominin lineages, genetic changes reveal and can be related to their migration patterns, habitats and ultimately archeological records (Skoglund and Mathieson 2018).

Generally, comparative genomics between great apes and human lineages exposed several classes of genetic changes, ranging from gross chromosomal rearrangements, allelic losses and duplications to single nucleotide variants (SNVs), all of them affecting both non-coding enhancers, and RNA/protein sequences (Pollen et al. 2023). In consequence, these can be classified as either affecting gene expression timing, pattern, or dosage by mutations in copy number variation or cis-regulatory elements, or as modifications in molecular functions through amino acid changes.

Genetic data has a major advantage over other methodologies. It allows establishing robust models to impute ancestral genomic states and branches along phylogenies. This is more difficult for approaches driven by archaeology or comparative neuroanatomy, which are primarily comparative (and not yet formalized) to impute ancestral structural or functional brain states. Likewise, as introduced above, one is not primarily interested in evolutionary changes in brain regions or behaviors per se, but actually in those that are under evolutionary pressure and contribute to the speciation and/or adaptation. This implies genetic changes under adaptive selection, which eventually become fixed, unlike those that are eliminated from the population. To this end, genetic datasets can be mined for signatures of ongoing purifying or adaptive selection in form of the population dynamics of archaic versus modern alleles in AMH and ω-values (dN/dS) from non-synonymous (dN) versus synonymous (dS) codon substitutions (Dorus et al. 2004).

In recent years such approaches were extremely successful in mapping out evolutionary important structural and genetic variants. The key challenge is to infer the functional consequences of these variants at the level of phenotypic adaptation (Figure 1). In cases where the mutation(s) introgressed into the AMH population, specific functional consequences can be directly predicted from the rich source of human genome or phenome wide association studies (G/PheWAS). At the neurocognitive level, these data associate Neanderthal alleles with mood disorder and depression (Simonti et al. 2016). If the archaic mutations and alleles did not segregate into the modern human populations, the functional implications of the phenotypic adaptations can be estimated by looking at overrepresentation of these genes in specific functional domains in the gene ontology (GO) or human phenotype ontology databases (Castellano et al. 2014). Interestingly, this approach dissociated functional selection for skeletal morphology during Neanderthal speciation from traits in the AMH lineage, which were associated with hyperactivity and aggressive behavior (e.g. adenylosuccinate lyase (ADSL), glycine dehydrogenase (GLDC), and SLITRK1) (Castellano et al. 2014). An additional in-depth meta-survey of available phenotypic predictions for archaic (Denisovan and Neanderthal) alleles converged on skeletal and facial features, skin physiology, inflammation, and disease susceptibility (such as COVID) for somatic domains (Brand et al. 2022). Moreover, in cognitive behavioral domains the archaic alleles are associated with addiction, schizophrenia, exploration, and aversive responding, decreased empathy, dopamine signaling, chronotype, mood disorder and depression. In addition to SNVs, phenotypic differences can be also caused by secondary effects on gene expression patterns. Such effects are mediated by remodeling spatio-temporal patterns of gene expression at cell types and systems levels in development and adult brains, underlying and contributing to the phenotypic evolution (Halligan et al. 2013).

At the level of gene expression, large scale in silico approaches aim to identify cis-regulatory elements in the archaic alleles via the Encyclopedia of DNA Elements (ENCODE) (The ENCODE Project Consortium 2012) or in the brain cell types *in vivo* (Chan et al. 2023). Together with the expanding phylogenetic transcriptomics in cell types and brain regions across mammalian brains, this will allow to compare gene expression differences in extant species and potentially infer ancestral states (McKenzie et al. 2018; Wei et al. 2022). Indeed, there is considerable evolution in mammalian brain gene expression, which in turn underlies evolutionary selection pressures (Khaitovich et al. 2006). Charting databases (e.g., genome-tissue expression consortia) that contain expression quantitative trait loci (eQTLs) allows to associate the effect of regulatory variants onto gene expression. Such approaches allowed to link specific gene sets to similar differential gene expression patterns and its functional consequences for neurological processes (Dannemann et al. 2017). Thus, there are in principle functional consequences that can be predicted for those alleles present in the human population. However, beyond comparative analyses there are no standardized methods to impute ancestral states of genetic regulation in adaptations specific to extinct species in human phylogeny. Only recently, such strategies have been successfully employed to mine for expression differences in archaic brains (PrediXcan) (Colbran et al. 2019). This regression models, trained on extant GTEx data of extant species, allows to predict regulatory differences and divergently regulated genes in human phylogeny. Functions associated with these differences can be then compared with biobank data linked to electronic health records. Such an approach has identified genes involved in supraorbital ridge development, immune, cardiovascular diseases and mental disorders (Colbran et al. 2019).

For any of the phenotypic predictions above, the next logical step is supporting genetic data with the underlying physiological mechanism. This can be achieved by identifying evolutionary important genes from genetic candidate screens (experimental screening or in silico evolutionary genetic identification) and linking them to function by performing mechanistic analyses in suitable model systems. These workflows yielded several landmark discoveries in two hallmarks of human evolution: neocortical expansion and functional evolution.

Several studies explored human neocortical expansion starting with targeted expression profiling of progenitor lineages in fetal AMH brains, identification of the human specific gene ARHGAP11B (Florio et al. 2015). This gene has a human-lineage specific duplication and is thus genetically associated with increased brain size at birth. To test this hypothesis experimentally, the gene was heterologously expressed in mice (Florio et al. 2015; Xing et al. 2021), ferrets (Kalebic et al. 2018) and then in marmoset models (Heide et al. 2020). These ‘humanized’ animals allowed for a specific neurodevelopmental characterization. Indeed, the data demonstrate that this gene increases brain size via amplification of a specific neuronal progenitor pool in the developing human brains (Florio et al. 2015). Moreover, a similar tissue targeted expression screening method also identified the AMH Transketolase-like 1 (TKTL1) allele to be involved in this phenomenon (Pinson et al. 2022). Gain of function experiments showed marked amplification of neurons in the frontal lobe in mouse and ferret cortex. This data, supported by loss of function experiments in AMH organoids suggests that AMH have more frontal neurons than Neanderthals, despite possessing a rather similar sized neocortex. This is suspected to have evolved by increasing proliferation of the neuronal progenitor pool specifically in frontal areas (Pinson et al. 2022).

At the brain functional level, the *FOXP2* gene is perhaps the most iconic example. FOXP2 was originally identified in language disorders in AMHs. Interestingly, it then emerged as one of the genes with the highest signature of positive selection from comparative genomics in great apes and AMH, suggesting a link to evolution of human specific speech and language skills. Complementing this with functional genetic data, expressing human versus great ape alleles in mouse models, indeed revealed altered vocalization possibly resulting from remodeled cortico-striatal circuit function as a possible underlying mechanism (Enard et al. 2009).

Recent revolutions in comparative genomics and comparative brain-wide single cell transcriptomics across primate and mouse models are about to dramatically deepen our understanding of the genetic pattern of human brain evolution (Garin et al. 2022). Moreover, molecular engineering in mouse-primate-human iPSC lines and brain organoids models now allow for functional genetic screens to explore molecular and cellular effects of evolving genes (Kanton et al. 2019; Trujillo et al. 2021) and their phenotypic contribution (Dannemann et al. 2020). Linking these technologies enables workflows for a large-scale identification and mechanistic dissection of human evolution from molecular to cellular levels that can be probed in genetically engineered rodent and primate models (Pollen et al. 2023; Reiner et al. 2021).

## 4 Mesoscale computational neurogenetic approaches for correlative archeology of mind

The success of bottom-up molecular/cellular experimental approaches will deepen and greatly expand the mechanistic insights of brain evolution in the future. However, there is not only a need but also an opportunity to link these evolutionary molecular/cellular mechanisms to systems-level data on behavior and cognitive traits (currently inferred from archeology) for the correlative archeology of mind (Figure 1). Similarly to correlative imaging (Walter et al. 2020), correlative archeology gathers information from several complementary modalities in order to create a composite and integral view, linking together multi-modal exploration across biological scales, ranging from archeological findings, animal and organoid models to genetic variants. As for behavioral neuroscience (Pfaff et al. 2019), correlative strategies fusing genetic data with circuit level dynamics and behavioral data should deepen our mechanistic understanding of evolutionary processes.

Importantly, such workflows will allow tracing of multi-genic effects emerging across functionally coupled brain networks, that contribute to higher cognitive functions. Critically, they can identify and differentiate between the specific human cognitive domains which are otherwise very difficult to capture and dissociate with molecular/cellular level mechanistic methods or archeological inferences alone. Ultimately, such approaches will facilitate work aiming to retrace multi-genic traits over time and compare the effects across a full spectrum of human brain functions and cognitive tasks (Matoba et al. 2022; Schlag et al. 2022; Sha et al. 2021).

Initially, predictions of multi-genic evolutionary features compared human versus Neanderthal alleles to skull brain shapes with differential globularization (Neubauer et al. 2018) and neuroanatomical features (regional gray-white matter volume, sulcal depth, gyrification index). The data allowed to identify specific brain regional adaptations in interparietal sulcus and visual cortex at the structural level (Gregory et al. 2017). Furthermore, the Neanderthal-derived genetic variations could then be related to specific differences in Neanderthal to AMH functional network connectivity of these regions. This approach supports the finding of altered visual processing as trait diverging between archaic and AMHs (Gregory et al. 2021).

Recently, several approaches started combining functionally linked genetic data with brain genetic makeup (specific expression sites) to retrieve mesoscale brain networks. These networks were then correlated to existing known functional networks obtained from cellular circuit physiology and regional fMRI approaches (Ganglberger et al. 2018; Richiardi et al. 2015), ultimately relating multi-genic effects to specific functional cognitive tasks. In an evolutionary setting, it is possible to impute ancestral states of functional networks from such mesoscale approaches, using multi-level brain data for evolutionary insight (Wei et al. 2019; Zwir et al. 2022). Comparative analysis of human with chimp and macaque used functional imaging and transcriptomics to focus on cognitive brain networks, demonstrating enrichment of gene sets having heightened divergence in the human lineage, particularly in higher-order cognitive networks. This phenomenon was particularly seen in the default mode network, a network that is essential for social cognition or mental self-reflections and is distributed across rapidly expanding cortical regions. The human-enriched genes were related to synaptic function, neuronal connectivity, neuronal development and autism spectrum (Wei et al. 2019). Evolutionary important genes were functionally associated with emotional reactivity, self-control, and self-awareness, where humans have more unique sequences compared to chimp and Neanderthal. Interestingly, many human-specific SNPs were identified as non-coding regulatory sequences and were found to be co-expressed in brain regions linked to emotions, self-awareness and autobiographical memory (Zwir et al. 2022).

A recent computational study used such mesoscale integrative approaches to project multi-genic genomic selection in 60 Mya of AMH phylogeny directly to a high-resolution neuroanatomical brain atlas and functional networks across cognitive domains. This was achieved by fusing an array of archaic genomes, cellular and regional level brain gene expression data and human task fMRI. This reconstructed traces of adaptive selection across cognitive traits and enabled a holistic view of evolutionary forces across diverse cognitive functions (Kaczanowska et al. 2022). This approach successfully pinpointed both language and strategic thinking as the most important adaptive selections emerging in AMH after splitting from its common hominin ancestors with other archaic hominins (Neanderthal and Denisovan). Moreover, adaptive selection was prominently bound to excitatory cells and via genes associated with brain size and synaptic functions and higher cognitive traits (e.g., mathematical skills) and psychiatric conditions (e.g., autism, schizophrenia). Thus, this mesoscale computational neuroarcheology approach ‘correlates archeology’ from molecular/cellular mechanics of brain size expansion and synaptic physiology to evolutionary selection of language and strategic thinking to archeological records on social cultural revolution (Figure 1).

From here on, one clear goal for any computational neuroarcheological approaches will be to improve the inference of ancestral states in standardized ways by integrating the ever-expanding data sets in the mouse to AMH lineage. This will require integrating ancestral projections from top-down archeological and fossil interpretations (such methods are being employed for brain size and gross anatomical organization) (Miller et al. 2019), archeology related data (Foley 2016) and comparative genetic and cell type profiling (Pollen et al. 2023). Similarly, these approaches should gain predictive power by integrating large scale neuronal recordings and brain functional imaging across rodent and primate model species. Jointly, combining these methodologies will improve imputing ancestral states and branch-wise evolution with greater precision and further into the past. Ultimately, this generates a successively refined evolutionary landscape of the human mind that links molecular mechanics to cognitive traits and archeological traces (Figure 1).

Given the complexity of multi-level brain science (Pfaff et al. 2019), this quest will require organizing the current fractionated and competing efforts from evolutionary geneticists, organoid modelers, neuroscientists and archeologists into a coherent joint and integrative exploration of the human past.
